# PPAR-*α* Ligands as Potential Therapeutic Agents for Wet Age-Related Macular Degeneration

**DOI:** 10.1155/2008/821592

**Published:** 2008-04-06

**Authors:** Marisol del V Cano, Peter L. Gehlbach

**Affiliations:** The Wilmer Ophthalmological Institute, School of Medicine, Johns Hopkins University, Baltimore, MD 21287-9278, USA

## Abstract

The peroxisome proliferator-activated receptors (PPAR's) are members of the steroid/thyroid nuclear receptor, superfamily of transcription factors. There are currently three known PPAR subtypes, *α*, *β*, and *γ*. The PPARs are now recognized participants in a number of biological pathways some of which are implicated in the pathogenesis of age-related macular degeneration (AMD). These include immune modulation, lipid regulation, and oxidant/antioxidant pathways important to the onset and progression of “dry” AMD, and vascular endothelial growth factor (VEGF) mediated pathways that stimulate choroidal neovascularization (CNV), characteristic of “wet” AMD. PPAR-*α* is found in retina and also on vascular cells important to formation of CNV. At this time, however, relatively little is known about potential contributions of PPAR-*α* to the pathogenesis of dry and wet AMD. This review examines current literature for potential roles of PPAR-*α* in the pathogenesis and potential treatment of AMD with emphasis on prevention and treatment of wet AMD.

## 1. INTRODUCTION

Age-related macular degeneration (AMD) is the leading cause of new blindness in the Western World and is currently responsible for more than half of all legal blindness in the United States. There are approximately 8 million people in the U.S. with early or intermediate stage AMD. Approximately one million of these people will develop advanced disease within the next five years [1–5]. Currently AMD is estimated to affect about 50 million people worldwide. With aging of the population this number is expected to double by the year 2020. Strategic approaches to management of AMD include delaying onset and progression of nonneovascular “(dry)” disease; preventing conversion from dry to wet disease and treatment of wet disease.

While specific antioxidant vitamin formulations are now known to delay progression of intermediate disease, current treatment of AMD focuses largely on providing therapeutic intervention following the progression of intermediate “(dry)” disease to late stage “(wet)” disease. The neovascular (“wet” or “exudative”) form of AMD can lead to rapid visual decline and accounts for nearly 90% of vision lost. It is characterized by development of pathologic choroidal neovascularization (CNV). Early strategies to ablate CNV used thermal laser or photodynamic therapy. These are now less frequently used as treatments that antagonize the effects of vascular endothelial growth factor (VEGF), continue to enhance efficacy, and improve outcomes. Currently pegaptanib, ranibizumab, and bevacizumab are considered relatively safe and achieve therapeutic effects that may include inhibition/regression of CNV, decreased vascular leakage, absorption of subretinal fluid, and improved vision [6–10].

The peroxisome proliferator-activated receptors (PPAR's) are not, at the present time, known as direct treatment targets in the management of AMD. Each represents a separate nuclear receptor of the steroid super-family of ligand activated transcription factors that induce steroid hormones, thyroid hormones, vitamin D, and retinoid acid receptor [11]. PPAR's comprise a family of three ligand-activated transcription factors (*α*, *β*, and *γ*) that are characterized by distinct function, ligand specificity, and tissue distribution. The PPAR transcription factors regulate transcription of many genes involved in differentiation, proliferation, and apoptosis, in a variety of cell types. During gene expression the PPAR forms a heterodimer receptor complex with the 9-cis-retinoic acid receptor (RXR). The PPAR/RXR heterodimer is associated with a multiprotein corepressor. When a ligand or agonist binds to the receptor, the corepressor complex dissociates. The heterodimer receptor then binds with peroxisome proliferator response elements on the promoter domain of target genes to stimulate transcription [12].

Three distinct PPAR's had been identified in mammals, PPAR-*α*, PPAR-*γ*, and PPAR-*δ* (also referred to as PPAR *β*). The first PPAR entity identified was PPAR-*α* agonist, which has multiple functions that result in an improved lipid profile, increasing high density lipoprotein cholesterol (HDL-C), decreasing triglycerides and free fatty acids, and shifting low density lipoprotein cholesterol (LDL-C) to larger less atherogenic particles. Each of these improvements in the lipid profile is potentially beneficial and may in theory lead to delay in AMD onset and progression thereby avoiding late stage or “wet” disease.

PPAR-*α* is transcribed from chromosome 22q12-13.1, is primarily expressed in tissues with elevated mitochondrial and peroxisomal fatty acids *β*-oxidation rates, such as liver, heart muscle, kidney, skeletal muscle, retina, and brown fat [13–15] and may have a potential role in oxidant/antioxidant pathways now strongly implicated in the pathogenesis of dry AMD. PPAR-*α* is also present in cells of the arterial wall associated with smooth muscle cells [16] and endothelial cells [17] and is found in monocytes and macrophages [18] that participate in CNV formation, characteristic of wet AMD [19]. The PPAR's are activated by a number of ligands including eicosanoids and fatty acids.In addition, synthetic antidiabetic and lipid lowering fibrates have been shown to activate PPAR-*γ* and PPAR-*α*, respectively. PPAR-*α* is the main target of fibrate drugs, a class of amphipathic carboxylic acids (gemfibrozil, fenofibrate, clofibrate) used in managing elevated triglycerides and cholesterol. PPAR-*γ* is highly expressed in adipose tissues and is a key mediator of adipogenesis [20, 21] and glucose homeostasis [22]. Little is known about the PPAR-*δ* which is expressed ubiquitously and has now been linked to obesity.

## 2. PPARs IN THE VASCULATURE

In addition to well established roles for the PPAR's in metabolic pathways, recent work suggests that the PPAR's may be involved in vascular regulation. Several groups have identified PPAR-*γ* and PPAR-*α* expression in monocytes/macrophages, vascular smooth muscle cells, and endothelial cells [16–18]. In the endothelium, PPAR-*γ* has been identified by PCR reaction [23], western blot and immunoprecipitation. PPAR-*α* has been demonstrated in the vascular endothelium by immunohistochemical technique [24]. While PPAR-*γ* has been widely studied for its antiangiogenic properties [25], recent studies now indicate that PPAR-*α* may have antiangiogenic properties as well [26, 27], a finding with potential therapeutic implications for wet AMD. PPAR-*α* agonists have recently been shown to inhibit expression of VEGF receptor 2 (VEGFR2) upregulation in neovascularization [26]. Varet et al. have demonstrated that fenofibrate, a PPAR-*α* ligand, inhibits angiogenesis in vitro and in vivo. They have also shown that fenofibrate reduces endothelial cell growth rate, endothelial cell mediated wound repair, and capillary tube formation. Interestingly fenofibrate has been shown to inhibit bFGF-induced angiogenesis in vivo [27]. Simultaneous inhibition of VEGFR2, bFGF, and VEGF would in theory have a profound effect on pathological angiogenesis in the eye.

PPAR-*α* and PPAR-*γ* are associated with anti-inflammatory and antioxidant activity [28–30] and have antiatherogenic effects [31]. Each of these pathways is considered important to the onset and progression of early AMD and to development of late choroidal neovascularization. PPAR-*α* activators inhibit expression of vascular cell adhesion molecules on the endothelium that are important for the development of new blood vessels and for atherogenesis [32]. Experimental evidence suggests that the PPAR activators prevent in vitro vascular muscle cell growth [33], limit inflammatory responses [16], and are proapoptotic indicating a potential role in vascular remodeling [34]. Such activity could theoretically inhibit the transition from dry to wet AMD. PPAR-*α* agonists also inhibit interleukin-1-induced production of interleukin-6 and prostaglandins [16]. Moreover, Delerive et al. have demonstrated prolonged inflammatory responses and increased interleukin-6 production in aortic explants of PPAR-*α* deficient mice [35] underscoring the anti-inflammatory potential of PPAR-*α*.

## 3. PPAR-*α* IN ANGIOGENESIS

Pathological angiogenesis leading to choroidal neovascularization is pathognomonic of “wet” AMD. Angiogenesis is the formation of new blood vessels from preexisting vessels and involves endothelial cell proliferation, migration, and organization into new capillary tubes. Pathological angiogenesis is integral to a number of prevalent ocular diseases characterized by the development of ocular neovascularization including but not limited to wet AMD, diabetic retinopathy, corneal neovascularization, the occlusive retinal vasculopathies, and retinopathy of prematurity. Inhibitors of ocular angiogenesis therefore have broad therapeutic implications for patients with these diseases.

Varet et al. demonstrated inhibition of angiogenesis by the PPAR-*α* ligand fenofibrate [27]. The antiangiogenic properties exhibited were characterized by a dose-dependent decrease in endothelial cell proliferation and apoptosis. Fenofibrates also reduced endothelial cell migration in vitro and capillary tube formation in a matrigel assay. Meissner et al. have also reported a reduction in endothelial cell proliferation, migration, and tube formation following treatment with fenofibrates and also with the PPAR-*α* agonist Wy14643 [26]. In further support of the evident antiangiogenic effect is the observation that several PPAR-*α* agonists decrease expression of VEGF receptor 2 (VEGFR2) in human umbilical endothelial cells (HUVECs) [26].

VEGFR2 is the most potent of the VEGF receptors. When activated VEGFR2 initiates signaling that leads to endothelial cell proliferation and also to expression of cytoprotective antiapoptotic molecules [36]. VEGFR2 is detectable only at relatively low levels in the adult vasculature; it is markedly up regulated by blood vessels during chronic inflammation, hypoxia, tumor growth, and wound repair. VEGFR2 and VEGF expression both increase as part of the angiogenic response and this coordinate response is observed in wet AMD as well as other ocular diseases characterized by pathological neovascularization [37, 38]. VEGF has been identified in fibroblastic cells and transdifferentiated RPE cells in surgically excised choroidal neovascular membranes (CNV) [39, 40]. VEGF expression is also increased in macular RPE cells in patients with AMD [41]. Vitreous VEGF levels are significantly higher in AMD patients with CNV as compared to healthy controls [42]. VEGF production is also increased in RPE cells, retinal vascular endothelial cells, retinal pericytes [43–45], and Muller cells [46]. The endothelial cells of the retinal vasculature possess numerous high-affinity VEGF receptors.

PPAR-*α* agonists have been associated with a reduction in VEGF levels in OVCAR-3 tumor as well as in DISS-derived ascites [47]. They also reduce microvessel density in these tumors. Other studies have similarly demonstrated that a reduction in PPAR-*α* message and activity is associated with hypoxia [48]. Hypoxia-induced VEGF expression contributes to choroidal and retinal neovascularization. The relative significance of the effect of PPAR-*α* on VEGFR2 and VEGF expression in the setting of AMD is not yet known.

## 4. PPAR-*α* AND WET AMD

Fenofibrates and other PPAR-*α* agonists are reported to decrease expression of VEGF and VEGFR2 that are central to the VEGF/VEGFR signaling cascade and important to the development of pathological CNV in AMD. Growth of experimentally induced CNV, via laser rupture of Bruch's membrane in a rat model, is inhibited by intravitreous treatment with a PPAR-*γ* agonist [49]. At the time of this writing, similar data has not been reported for PPAR-*α*. Evaluation of this question is however supported by evidence of PPAR-*α* reduction of VEGFR2 expression in endothelial cells [26] and reported decreases in tissue VEGF levels [47]. PPAR-*α* activators have also been shown to limit the expression of vascular cell adhesion molecules in the endothelium, an early step in atherogenesis and an important step in the development of new blood vessels [32]. Inhibition of CNV initiation and early progression of CNV are therefore theoretical benefits of PPAR-*α* agonist treatment. Described proapoptotic effects also suggest therapeutic roles in early CNV development or late regression of CNV [34].

With reports that the PPAR's limit inflammatory as well as oxidative responses and improve lipid profiles [16, 28, 29, 35, 37, 50], it is tempting to speculate on a potential role in delaying onset and progression of nonneovascular “(dry)” disease, thereby potentially preventing latter “wet” stages of disease. There is a substantial literature linking oxidative damage to dry AMD pathogenesis [51]. PPAR-*α* could theoretically inhibit AMD progression via effects on oxidative pathways. It has been previously reported that PPAR-*α* activation induces the expression and activation of antioxidant enzymes, such as super oxide dismutase and glutathione peroxidase [29]. It has also been reported that PPAR-*α* agonists are neuroprotective in the CNS, and that this neuroprotection has been associated with a decrease in cerebral oxidative stress. Consumption of direct acting antioxidants to provide protection to the retina and the RPE is supported by the AREDS clinical trial that has added antioxidant formulation to the routine care of dry AMD. Whether the antioxidant effects of PPAR-*α* activation are comparable to those of AREDS formulation is not known.

Because fenofibrates are orally administered and have an established safety profile in the treatment of atherosclerosis, investigations pertaining to the impact of oral therapy on oxidative stress, VEFGR2, VEGF, and CNV growth are important. It is also important to consider examining for potential beneficial effects on onset and progression of nonneovascular “(dry)” disease and conversion from dry to wet disease. These and other factors support a hypothesis that asks whether PPAR-*α* may play a therapeutic role in either prevention or treatment of wet AMD.

## 5. SUMMARY

AMD remains the leading cause of new blindness in people over 65 years of age and is the leading cause of new blindness in the Western World. The conversion of dry AMD to wet AMD is associated with most of the attendant visual decline. Currently a variety of antiangiogenic treatments directed at halting CNV growth and leakage are the mainstay of therapy. The most frequently injected agent ranibizumab (Lucentis) results in stabilization of visual acuity at the pretreatment level for a majority of patients and results in improvement of visual acuity by 3 or more lines in about 1/3 of those treated. The therapy does not however restore visual acuity to normal levels in the majority of those treated. Moreover, therapy with ranibizumab and other currently available VEGF antagonists requires frequent intravitreous injections and is associated with significant expense, some risk, and for most, incomplete recovery of vision.

An oral therapy with an established safety profile that favorably modified VEGF/VEGFR signaling and increased the antioxidant capacity could significantly impact the therapy of wet AMD. Taken collectively, the PPAR's demonstrate favorable biological activity in pathophysiological pathways relevant to the onset and progression of nonneovascular and neovascular age-related macular degeneration. The relative importance of the PPAR-*α* pathway in AMD is not yet known. There is, however, sufficient preliminary evidence to support further study of a potential role for PPAR-*α* pathway modulation as an adjuvant or primary treatment in AMD.
